# Manual lymphatic drainage for lymphedema in patients after breast cancer surgery: A systematic review and meta-analysis of randomized controlled trials

**DOI:** 10.1097/MD.0000000000023192

**Published:** 2020-12-04

**Authors:** Mining Liang, Qiongni Chen, Kanglin Peng, Lu Deng, Li He, Yongchao Hou, Yang Zhang, Jincai Guo, Zubing Mei, Lezhi Li

**Affiliations:** a Clinical Nursing Teaching and Research Section, the Second Xiangya Hospital, Central South University, Changsha, Hunan Province; b Department of Psychiatry, the Second Xiangya Hospital; c Mental Health Institute of the Second Xiangya Hospital, Central South University; d Chinese National Clinical Research Center for Mental Disorder (Xiangya), Chinese National Technology Institute on Mental Disorders, Hunan Key Laboratory of Psychiatry and Mental Health, Changsha, Hunan, 410011; e Emergency Department, Shanxi Provincial People's Hospital, Taiyuan 030001, Shanxi; f Nursing Teaching and Research Institute, Medical College of Guangxi University of Science and Technology, Liuzhou, Guangxi Province, 545005; g Department of Medical Technology, Changsha Stomatological Hospital, Changsha, Hunan, 410006; h Department of Anorectal Surgery, Shuguang Hospital, Shanghai University of Traditional Chinese Medicine, Shanghai, 201203; i Xiangya School of Nursing, Central South University, Changsha, Hunan, 410011, Republic of China.

**Keywords:** breast cancer, lymphedema, manual lymph drainage, meta-analysis, randomized controlled trial

## Abstract

**Background:**

Studies have shown that manual lymphatic drainage (MLD) has a beneficial effect on lymphedema related to breast cancer surgery. However, whether MLD reduces the risk of lymphedema is still debated. The purpose of this systematic review and meta-analysis was to summarize the current evidence to assess the effectiveness of MLD in preventing and treating lymphedema in patients after breast cancer surgery.

**Methods:**

From inception to May 2019, PubMed, EMBASE, and Cochrane Library databases were systematically searched without language restriction. We included randomized controlled trials (RCTs) that compared the treatment and prevention effect of MLD with a control group on lymphedema in breast cancer patients. A random-effects model was used for all analyses.

**Results:**

A total of 17 RCTs involving 1911 patients were included. A meta-analysis of 8 RCTs, including 338 patients, revealed that MLD did not significantly reduce lymphedema compared with the control group (standardized mean difference (SMD): −0.09, 95% confidence interval (CI): [−0.85 to 0.67]). Subgroup analysis was basically consistent with the main analysis according to the research region, the publication year, the sample size, the type of surgery, the statistical analysis method, the mean age, and the intervention time. However, we found that MLD could significantly reduce lymphedema in patients under the age of 60 years (SMD: −1.77, 95% CI: [−2.23 to −1.31]) and an intervention time of 1 month (SMD: −1.77, 95% CI: [−2.23 to −1.30]). Meanwhile, 4 RCTs including, 1364 patients, revealed that MLD could not significantly prevent the risk of lymphedema (risk ratio (RR): 0.61, 95% CI: [0.29–1.26]) for patients having breast cancer surgery.

**Conclusions:**

Overall, this meta-analysis of 12 RCTs showed that MLD cannot significantly reduce or prevent lymphedema in patients after breast cancer surgery. However, well-designed RCTs with a larger sample size are required, especially in patients under the age of 60 years or an intervention time of 1 month.

## 1 Introduction

Lymphedema is a common complication after breast cancer surgery. It is characterized by persistent tissue swelling due to abnormal accumulation of lymph in tissues.^[1]^ Lymphedema has a significant negative physical and psychological impact on individuals. It affects about 15% to 30% of patients after breast cancer surgery.^[2–4]^ Therefore, there is an urgent need to implement effective methods to treat or prevent lymphedema.

Physical therapy, such as manual lymphatic drainage (MLD), is a common treatment for lymphedema related to breast cancer. MLD is carried out by a trained MLD therapist, who uses specialized and gentle hand movement to give a pumping action on the skin without oils. This technique is a type of skin massage, which improves lymph flow and reabsorption without increasing capillary filtration. As a result, MLD reduces the tissue swelling and softening fibrosis in the trunk and arm.^[5,6]^ MLD can not only be implemented alone, but can also be implemented with other therapies, or as a part of Complete Decongestive Therapy (CDT). CDT contains the 4 following components: MLD, bandaging, exercises, and skin care practices.^[7]^ Bandaging involves a compression bandage, which aims to reduce swelling by reducing of fluid formation and the amount of lymph reflux.^[7]^ Exercises combine compression with active and repetitive movements in the affected area of the body, which can promote lymph flow and reduce swelling.^[1]^ Physiotherapists teach skin care exercises to avoid skin infection.^[1]^

So far, researchers have investigated the effect of using MLD on the treatment and prevention of lymphedema related to breast cancer surgery. Although some studies have shown a positive difference between MLD and other therapies for lymphedema, other studies have inconsistent results.^[7–11]^ In addition, some studies have shown that MLD has a greater impact on reducing the incidence of lymphedema; nevertheless, other studies do not find such association.^[12,13]^ Two systematic review and meta-analysis, conducted by Ezzo et al and Huang et al, respectively, stated that a systematic analysis of previous research reveals little distinction in the treatment or prevention of lymphedema related to breast cancer by comparing MLD with other interventions.^[1,14]^ Furthermore, the results provided limited data as a result of a small sample size of the included studies.^[1,14]^ Therefore, an updated and comprehensive meta-analysis of all RCTs is needed to evaluate the effect of MLD on the treatment and prevention of lymphedema in patients after breast cancer surgery.

## 2 Methods

This study has been conducted and reported in line with PRISMA (Preferred Reporting Items for Systematic Reviews and Meta-Analyses) and AMSTAR (Assessing the methodological quality of systematic reviews) Guidelines. No ethical approval is needed for this network meta-analysis, because we used published data.

### 2.1 Data sources and search strategy

This systematic review and meta-analysis were performed in accordance with the 2009 Preferred Reporting Items for Systematic Reviews and Meta-analysis (PRISMA) guidelines.^[15]^ PubMed, EMBASE, and the Cochrane Library were systematically searched from their inception until May 2019 with no language limitation to identify all relevant randomized controlled trials (RCTs). A manual screen of reference cited in the previous published studies, reviews, and meta-analyses were also conducted to identify potentially missed articles. Two authors (ML and QC) independently searched the past studies using the following medical subject heading (MeSH) terms and free text words: breast neoplasms, lymphedema, physical therapy modalities, drainage (as MeSH terms), combined with breast/mammary, cancer∗/tumor∗/tumour∗/carcinom∗/neoplas∗/malignan∗/adenocarcinoma, lymphoedema/lymphedema/oedema /edema/swelling/elephantias∗/“lymphatic edema,” “manual lymph∗” drainage/physiotherapy/“sequential pneumatic compression”/“complex decongestive therapy”/“decongestive lymphatic therapy”/“Foldi method”/“Vodder method” (as free text in the title or abstract). Detailed search terms and literature search strategies are provided in Supplementary Appendix 1.

### 2.2 Study selection

Two reviewers (ML and QC) independently screened and identified all retrieved records by reading the titles and abstracts for potential eligible articles. We used EndNote X7 software to perform data management. Disagreements were resolved by requiring another senior author (HL or LL) to screen articles until a consensus was reached.

The RCTs were included in this systematic review and meta-analysis if they satisfied the following criteria:

(1) Type of study: randomized controlled trial (RCT);
(2) Study subjects;
(3) Study methods: RCTs enrol breast cancer patients who are receiving MLD, describe the definition of lymphedema, and provide the inclusion and exclusion criteria for enrolling participants;
(4) Intervention: The experimental group received MLD, while the control group received compression bandaging and other methods (such as physical therapy, simple lymphatic drainage (SLD), etc) for treatment;
(5) Main outcomes: RCTs evaluate the severity of lymphedema or the incidence of lymphedema. RCTs provide risk ratio (RR) estimate and its 95% confidence interval (CI) or an arm volume reduction comparing the MLD group with the control group.

Exclusion criteria included: review article, comments, meta-analysis, studies without related outcomes, and studies without RCT design. When there were duplicated publications of the same study, we referred to one of the studies that provided the most informative data.

### 2.3 Data extraction and quality assessment

Two reviewers (ML and LH or LD) independently extracted data from all relevant articles. They extracted the following information from each study: first author, publication year, study country, study design, inclusion and exclusion criteria, definition of lymphedema, assessment of lymphedema, sample size, mean age, outcome, and follow-up period. Discrepancies were discussed by a third reviewer until consensus was reached.

The quality of the studies was evaluated by the following criteria:

1) study design,
2) data analysis,
3) adequacy of the randomization,
4) allocation concealment,
5) adequacy of blindness, and
6) number of drop-outs.

### 2.4 Outcomes assessments and definition of lymphedema

The effect of MLD on the prevention of lymphedema was evaluated by the incidence of lymphedema, and the efficacy of MLD in the treatment of lymphedema was assessed by the percentage reduction in total of lymphedema from baseline to follow-up period. The volume of the arm was measured by submerging the affected and unaffected arm in a container with water and the volume displacement was measured in millilitre. The arm volume with circumferential measurement was marked in 4 cm increments up the arm from the ulnar styloid to the axilla. The definition of lymphedema is an increase of more than 10% in volume between the abnormal and normal arm; a difference of more than 200 ml in arm volume or more than 20 mm in the circumference between the abnormal and normal arm.

### 2.5 Statistical synthesis and analysis

Statistical analysis was conducted using Stata Statistical Software (Version 12.0; Stata Corporation, College Station, TX) by 2 reviewers (ML and ZM). The reported CI limits, standard error, or range values were used to estimate the standard deviation when needed. The RR was calculated as the effect size of binary variables, and the standardized mean difference (SMD) was calculated as the effect of continuous variables. The summary RRs with corresponding 95% CIs were aggregated by the DerSimonian and Laird random-effects model.^[16]^

The Cochran's *Q* and *I*^2^ statistic was used to examine the interstudy heterogeneity, with an *I*^2^ value of more than 50%, indicating substantial heterogeneity. In addition, a subgroup analysis was performed to examine the potential sources of interstudy heterogeneity by analysing the possible basic variables such as research region, publication year, sample size, type of surgery, statistical analysis method, mean age, and intervention time. Publication bias was investigated visually by inspecting funnel plots and statistically by using Egger's as well as Begg's regression model.^[17,18]^ Furthermore, Duval's nonparametric trim and fill procedure was used to adjust the pooled estimates of potential unpublished studies when publication bias existed. *P* < .05 indicated statistical significance.^[19]^ We also conducted a sensitivity analysis to explore the influence of each study on the separate analyses of the studies.

## 3 Results

### 3.1 Search and selection of studies

Of the initial 413 eligible articles, 27 were considered to be potentially relevant studies for further review. After removing 10 studies, 17 studies met our inclusion criteria and were involved in this systematic review and meta-analysis. Figure A1 describes the process of study selection.

### 3.2 Study characteristics

Table 1 provides the characteristics of the 17 final included studies, including 1911 patients (range from 12 to 500) and were published in English journals from 1998 to 2018.^[7–13,20–29]^ Among those RCTs, 2 were conducted in North America, 1 in South America, 11 in Europe, 2 in Asia, and 1 covered multiple continents. Of the 17 included primary studies, 12 studies investigated the effect of MLD on the treatment of lymphedema and 5 studies reported the effect of MLD on the prevention of lymphedema. All patients underwent breast cancer surgery, ranging from 25 to 85 years of age.

**Table 1:** Characteristics of the included studies on the effect of MLD on preventing or managing breast cancer-related lymphedema.

Author	Year	Region	Inclusion criteria	No. of participants	Mean/median age (yr)	Control	Intervention	Follow-up period (mo)
Tambour	2018	Denmark	Lymphedema symptoms: >20 mm difference in circumference between the 2 arms	C: 35 I: 38	C: 60.9 ± 10.8 I: 62.9 ± 11.5	C: skin care + compression bandaging + activity guidance, 30 min/d twice/wk for 1 mo	I: C + MLD 60 min/d twice/wk for 1 mo	6
Devoogdt	2018	Belgium	A unilateral axillary dissection levels I, I–II or I–II, patient after breast cancer surgery	C: 81 I: 79	C: 55 ± 11 I: 56 ± 13	C: guidelines + exercises twice/wk, gradually diminished to once/2 wk for 6 mo	I: C + MLD, 1–3 times/wk, decreased to once/wk for 6 mo	60
Zhang	2016	China	Undergoing modified radical mastectomy patient after breast cancer surgery	C: 500 I: 500	C: <50:272 I: <55:266	C: educational strategy + exercise, 15 min/session, 3 sessions/d for 6 mo	I: C + MLD, 30 min/session, 3 times/d, from 10 to 30 d after surgery	12
Cho	2016	South Korea	Patient after breast-cancer surgery	C: 20 I: 21	C: 50.7 ± 9.6 I: 46.6 ± 6.8	C: physical therapy, 3 times/wk, for 4 wk	I: C + MLD, 30 min/time, 5 times/wk for 4 wk	NR
Bergmann	2014	Brazil	Lymphedema symptoms: >30 mm difference in circumference between the 2 arms	C: 29 I: 28	C: 63.6 ± 11 I: 62.2 ± 9.1	C: soft touch + skin care + compressive bandaging + remedial exercises, 3 times/wk, 24 d	I: C + MLD, 3 times/wk, 24 d	NR
Ridner	2013	USA	Lymphedema	C: 15 I: 15	C: 66.4 ± 11.3 I: 66.0 ± 10.2 Being age 21 or older	C: compression bandaging + 20 min of low-level laser therapy, 10 sessions	I: C + 20 min of MLD, 10 sessions	NR
Zimmermann	2012	Germany	Patient after breast-cancer surgery	C: 34 I: 33	C: 58.6 ± 12.2 I: 60.3 ± 8.2 (34–81)	C: exercises + chest physical therapy + self-drainage	I: C + MLD, 5 times/wk for 2 wk, then twice/wk from day 14 to 6 mo	NR
Belmonte	2012	Spain	Lymphoedema, more than 6 mo without manual lymphatic drainage treatment	G1: 18 G2: 14	G1: 69.6 ± 10.1 G2: 65.5 ± 12.7	G1: compression garments + exercises + skin care + electrotherapy, 5 d/wk for 2 wk, then no treatment for 1 mo, then MLD, 5 d/wk for 2 wk	G2: garments + exercises + skin care + MLD, 5 d/wk for 2 wk, then no treatment for 1 mo, then electrotherapy, 5 d/wk for 2 wk	NR
Devoogdt	2011	Belgium	Patient after breast-cancer surgery	C: 81 I: 77	C: 54.5 ± 11.1 I: 55.8 ± 12.5	C: guidelines + exercises 30 min/session, 2 times/wk, then decrease to 1 time/wk	I: C + MLD, 30 min/session, 1–3 times/wk, then decrease to once/wk for 40 sessions	12
Szolnoky	2009	Hungary	Lymphedema >12 mo after surgery	G1: 13 G2: 14	G1: 54.8 G2: 56.6	G1: MLD 60 min/d, 5 d/wk for 2 wk	G2: MLD 30 min/d then SPC 50 mm Hg 30 min/d, 5 d/wk for 2 wk	2
Didem	2005	Turkey	2–50 mm circumference difference between 2 arms, lymphedema with a duration of at least 1 yr	C: 26 I: 27	C: 60.5 ± 8.1 I: 57.7 ± 7.0 31–76 yr	C: bandging + elevation + exercise, once/d, 3 d/wk for 4 wk	I: C + MLD, once/d, 3 d/wk for 4 wk	NR
McNeely	2004	Canada	Lymphedema symptom: difference in volume of 150 ml between the 2 arms	C: 21 I: 24	C: 63 ± 13 I: 58 ± 13	C: compression bandaging, 45 min/d, 5 d/wk for 4 wk	I: C + MLD 45 min/d, 5 d/wk for 4 wk	NR
Williams	2002	UK	>10% volume difference between the 2 arms	G1: 15 G2: 16	G1: 59.7 ± 2.1 G2: 59.3 ± 2.4	Group 1: 3 wk of MLD, then 6-wk non-treatment, then 3 wk of SLD	Group 2: 3 wk of SLD, then 6-wk non-treatment, then 3 wk of MLD	NR
Sitzia	2002	UK	Lymphedema symptoms: percentage excess volume (PCEV) ≥ 20% in the affected arm	G1: 13 G2: 15	G1: 75 ± 10.2 (59–91) G2: 68 ± 10.8 (48–85)	G1: SLD 20 min + compression bandage + exercises, 5 d/wk for 2 wk	G2: MLD 40–80 min + compression bandage + exercises, 5 d/wk for 2 wk	NR
Andersen	2000	Denmark	Lymphedema symptoms: a difference in volume of 200 ml or circumference of 20 mm between the 2 arms	C: 22 I: 20	C: 56 (29–77) I: 53 (25–73)	C: sleeve-and-glove compression 32–40 mm Hg + skin care + safety precaution + exercises	I: C + MLD, 8 times for 2 wk	NR
Johansson	1999	Sweden	Lymphedema symptoms: >10% difference in volume between the 2 arms	C: 18 I: 20	C: 64 ± 12 (37–83) I: 58 ± 12 (41–80)	C: bandage compression for 3 wk	I: C + MLD for 5 d at last week	NR
Johansson	1998	Sweden	Lymphedema symptoms: >10% difference in volume between the 2 arms	G1: 12 G2: 12	G1: 57.5 (47.5–69.5) G2: 64 (52.5–69.5)	C: sleeve compression for 5 d/wk for 2 wk + SPC 40–60 mm Hg 2 h/d, 5 d/wk for 2 wk	I: Sleeve compression for 5 d/wk for 2 wk + MLD 45 min/d, 5 d/wk for 2 wk	NR

C = control group, G1 = group one, G2 = group two, I = intervention group, MLD = manual lymph drainage, NR = none report, PCEV = percentage change in excess limb volume, SLD = simple lymphatic drainage, SPC = sequential pneumatic compression, UK = United Kingdom.

Among the 17 RCTs, the intervention group and the control group were comparable for age and number of participants (Table 1). The majority of the studies conducted MLD using the Vodder method.^[8,11,24,26,27,29]^ Some studies applied MLD following Földi's technique.^[7,22]^ All of the participants received MLD by trained lymphedema physiotherapists who were experienced in administering all treatment sessions. Standard treatment in the control group included the following components: compression bandage or garments, education information for skin care, exercise guidance for reducing lymph flow, and safety precautions. Twelve studies compared the effect of MLD on the treatment of lymphedema related to breast cancer with other therapies. Across these 12 studies, 1 study compared the effects of low-level laser therapy with MLD,^[22]^ 1 study compared the effects of electrotherapy with MLD,^[9]^ 1 study compared the effects of sequential pneumatic compression with MLD,^[24]^ and 2 studies compared the effects of SLD with MLD^[10,27]^ on reducing arm volume in the affected arm. Five studies investigated the effect of MLD on the prevention of the incidence of lymphedema in patients after breast cancer surgery.^[21,23]^

### 3.3 Study quality evaluation

Table 2 presents the quality of the methodology used in the eligible studies. Of the 17 included studies, the methods of randomization for 8 studies were sufficient, the method of allocation concealment for 7 studies was acceptable, 6 studies used the assessor blinded method, and 1 study used the patient blinded method. In total, the percentage of loss to follow-up was less than 15% of all the elevated studies.

**Table 2:** Quality assessment of the included studies.

Study ID		Study design	Data analysis	Allocation generation	Allocation concealment	Blinding	Lost to follow-up
Tambour	2018	RCT	ITT	Adequate	Adequate	Assessor blinded	5.2% at 7 mo
Devoogdt	2018	RCT	ITT	Adequate	Adequate	Assessor blinded	1.2% at 6 mo
Zhang	2016	RCT	PP	Inadequate	Unclear	None reported	None
Cho	2016	RCT	ITT	Inadequate	Unclear	Assessor blinded	14.6% at 1 mo
Bergmann	2014	RCT	ITT	Inadequate	Unclear	None reported	13.6% at 24 d
Ridner	2013	RCT	PP	Computer-generated	Unclear	None reported	None
Zimmermann	2012	RCT	PP	Inadequate	Unclear	None reported	None
Belmonte	2012	RCT	ITT	Computer-generated	Adequate	Assessor blinded	11.1% at 2 mo
Devoogdt	2011	RCT	ITT	Adequate	Adequate	Assessor blinded	4% at 12 mo
Szolnoky	2009	RCT	ITT	Unclear	Unclear	None reported	None
Didem	2005	RCT	PP	Unmarked envelopes	Adequate	Patient blinded	5.4% at 1 mo
McNeely	2004	RCT	PP	Computer-generated code	Adequate	Assessor blinded	11.1% at 1 mo
Williams	2002	RCT	PP	Unclear	Unclear	None reported	6.5% at 3 wk
Sitzia	2002	RCT	PP	Computer-generated	Adequate	None reported	3.6% at 2 wk
Andersen	2000	RCT	ITT	Unclear	Unclear	None reported	2.4 at 3 mo, 9.5% at 12 mo
Johansson	1999	RCT	PP	Inadequate	Unclear	None reported	None
Johansson	1998	RCT	PP	Unclear	Unclear	None reported	None

ITT = intention-to-treat, PP = per-protocol, RCT = randomized controlled trial.

### 3.4 The effect of MLD on the treatment of lymphedema

**Figure 1:**
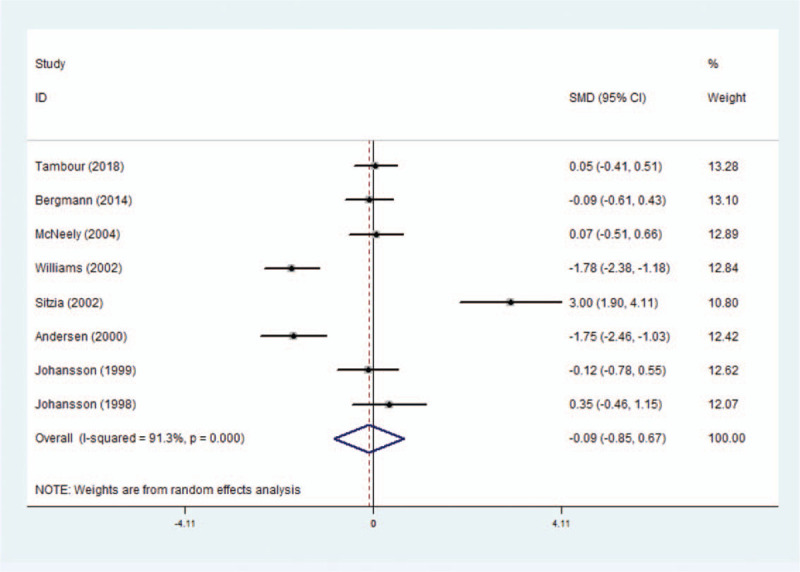
Forest plot for meta-analysis of the pooled SMDs of MLD treatment on post-mastectomy lymphedema in 8 RCTs.

In general, 12 eligible RCTs demonstrating the effect of MLD on treating lymphedema were published between 1998 and 2018 and had sample sizes ranging from 24 to 73 subjects. Among the 12 eligible RCTs, 8 RCTs were examined by carrying out a meta-analysis including 338 patients that used the same result evaluation indexes. The pooled SMD was −0.09 (95% CI: [−0.85 to 0.67]), which showed a non-significant effect of MLD on the treatment of lymphedema in patients after breast cancer surgery, and there was statistical interstudy heterogeneity (*I*^2^ = 91.3%; *P* < .001) (Fig. 1). Several subgroup analyses were carried out to explore the potential sources of interstudy heterogeneity on the estimated effect size.

An analysis of the region subgroups (Europe and America) showed that the heterogeneity was reversed and the effect of MLD on treating lymphedema was greater in the American subgroup (SMD: −0.02, 95% CI: [−0.41 to 0.37]; *I*^2^ = 0, *P* = .069) than in the European subgroup (SMD: −0.65, 95% CI: [−1.54 to 0.24]; *I*^2^ = 89.9, *P* < .001). Similarly, the heterogeneity declined when the included studies were stratified into subgroups based on the publication year, and this effect of the “publication year after 2010” subgroup (SMD: −0.01, 95% CI: [−0.35 to 0.34]; *I*^2^ = 0, *P* = .693) and the “publication year before 2000” subgroup (SMD: 0.07, 95% CI: [−0.44 to 0.58]; *I*^2^ = 0, *P* = .383) were greater than that of the “publication year between 2000 and 2010” subgroup (SMD: −0.16, 95% CI: [−1.88 to 1.56]; *I*^2^ = 95.7, *P* < .001). In addition, the heterogeneity declined when the included studies were stratified into subgroups based on the mean age subgroups (≧60 years and <60 years) and MLD could significantly reduce lymphedema in patients below the age of 60 years old (SMD: −1.77, 95% CI: [−2.23 to −1.31]; *I*^2^ = 0, *P* = .952). Furthermore, an analysis of the intervention time subgroups showed that the heterogeneity was reversed and MLD could significantly reduce lymphedema in patients when the intervention time was 1 month (SMD: −1.77, 95% CI: [−2.23 to −1.30]; *I*^2^ = 0, *P* = .952) (Table A5).

### 3.5 Publication bias and sensitivity analysis

**Figure 2:**
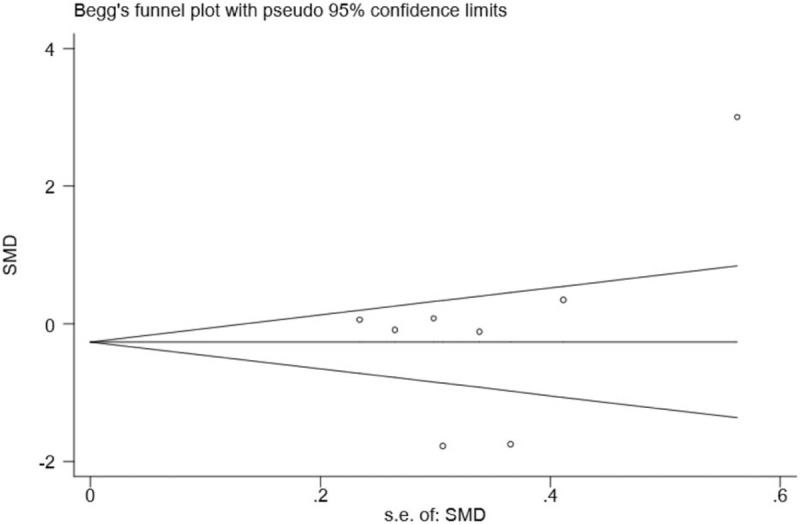
Funnel plot of the effect of MLD on the reduction of post-mastectomy lymphedema in 8 RCTs.

**Figure 3:**
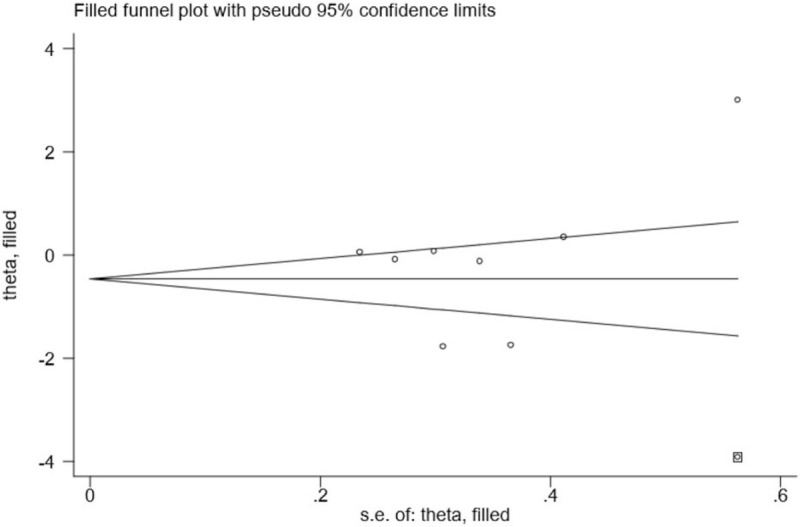
Duval's nonparametric trim and fill procedure for the effect of MLD on the reduction of post-mastectomy lymphedema in 8 RCTs.

**Figure 4:**
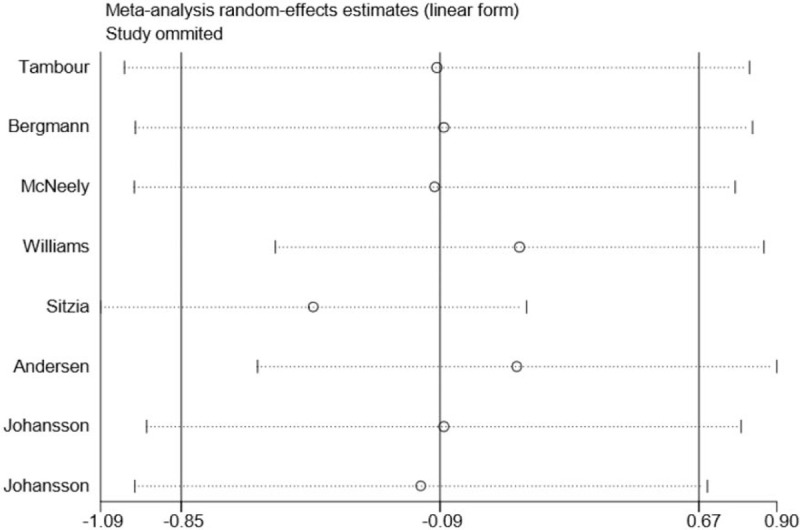
Sensitivity analysis for association between MLD use and the reduction of post-mastectomy lymphedema based on 8 RCTs.

Although substantial statistical heterogeneity was noted in this meta-analysis, almost all of the 8 included studies showed a similar direction of effect, thus demonstrating that some of the heterogeneity was mainly attributed to a variation in the magnitude of the estimated risk instead of the direction. No evident publication bias was identified when examining for funnel plot asymmetry with Egger's test (*P* = .445) or Begg's test (*P* = .711) (Fig. 2). However, due to the limited number of included studies, we should interpret this finding with caution. Trim and fill methods were conducted to analyse the sensitivity analysis and the results indicated 1 missing study in the funnel plot (Fig. 3). However, inputting this 1 hypothesized study did not largely alter the original pooled estimate SMD (−0.46, 95% CI: [−1.33 to 0.40]). Therefore, the results of this study were eligible and seemed to not be affected by publication bias. Moreover, we carried out a sensitivity analysis by excluding 1 trial each time and then recalculating the pooled SMD for the remaining trials to test the effect of each study on the overall estimates, which did not show an alteration of estimate when any one of the included trials was excluded (Fig. 4).

With regard to the remaining 4 studies, 142 patients demonstrating the effect of MLD on the treatment of lymphedema were not pooled in the meta-analysis. The data reported by Ridner et al and Szolnoky et al were not pooled because the method used to measure the change in lymphedema volume was a circumferential measurement.^[22,24]^ However, no statistically significant differences were found between the MLD treatment group and the group that combined MLD and low-level laser therapy in Ridner's study, and no significant differences were found between the MLD treatment group and the group that combined MLD with Intermittent Pneumatic Compression (IPC) at 1 and 2 months after the start of the therapy in Szolnoky's study.^[22,24]^ Furthermore, a study comparing the effects of MLD followed by low-frequency and low-intensity electrotherapy with the effects of low-frequency and low-intensity electrotherapy followed by MLD reported no significant difference in reducing lymphoedema volume after treatment.^[9]^ Moreover, Didem's study was not pooled as the instruments used to measure the lymphedema volume reduction are not reported.^[25]^

### 3.6 The effect of MLD on the prevention of lymphedema

**Figure 5:**
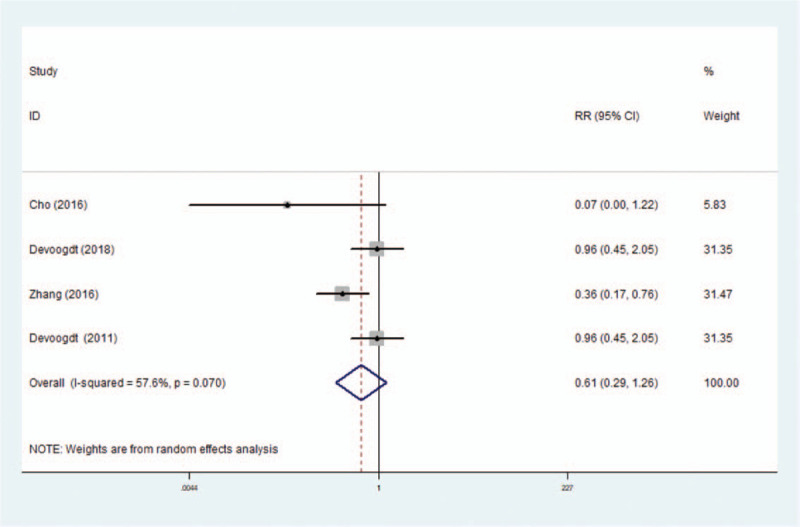
Forest plot for meta-analysis of the pooled RRs of MLD on risk of post-mastectomy lymphedema in 4 RCTs.

**Figure 6:**
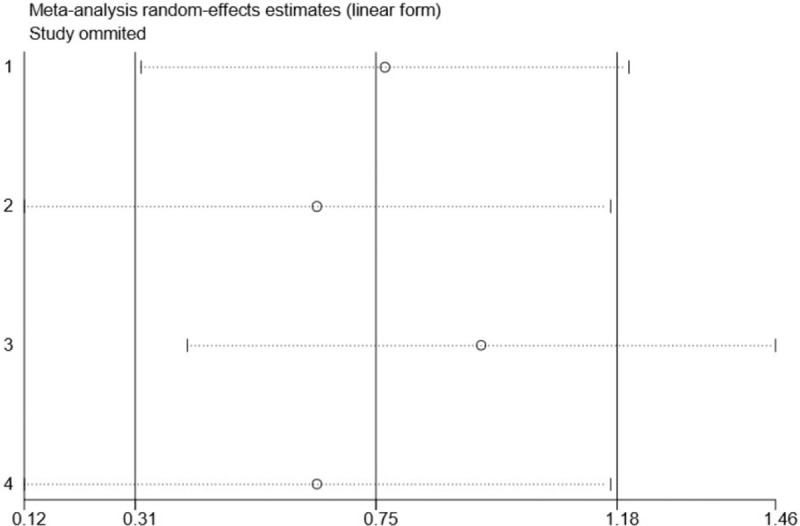
Sensitivity analysis for association between MLD use and risk of post-mastectomy lymphedema based on 4 RCTs.

Five RCTs including 1431 patients reported the effect of MLD on the prevention of lymphedema in patients after breast cancer surgery. We included 4 RCTs comprising 1364 individuals in meta-analysis that reported estimates of the RR for the risk of lymphedema with MLD use.^[12,13,20,21]^ We did not include the study of Zimmermann in meta-analysis as a result of their main outcomes used mean values of the arm volume measurements on the operated side. Zimmermann et al demonstrated that MLD applied immediately after breast cancer surgery prevented secondary lymphedema of the arm regardless of the surgery type at 6 months.^[23]^ The results of our meta-analysis showed that MLD could not significantly prevent the long-term risk of lymphedema (RR 0.61, 95% CI: [0.29–1.26]) (Fig. 5). In fact, we found that MLD could significantly prevent the risk of lymphedema in patients after breast cancer surgery within a 1-month period (RR 0.08, 95% CI: [0.01–0.61]) (Fig. A2). The results, however, should be interpreted with caution due to the limited number of studies. We also conducted a sensitivity analysis by excluding 1 RCT each time and then recalculating the pooled RRs for the remaining RCTs to test the effect of each study on the overall estimates. We did not find an alteration of the estimate when any one of the included RCTs was excluded (Fig. 6).

## 4 Discussion

### 4.1 Principal findings

The current systematic review and meta-analysis were conducted to assess the effectiveness of MLD on lymphedema after breast cancer surgery. The results of 8 RCTs that compared the effect of MLD with other therapies on the treatment of lymphedema related to breast cancer were summarized, which showed that MLD added no benefit in reducing the arm volume of the affected side. This estimated effect persisted in the analysis stratified by research region, publication year, sample size, type of surgery, statistical analysis method, mean age, and intervention time. The robustness effect was also confirmed by the trim and fill method and sensitivity analysis. Meanwhile, 4 RCTs comparing the effects of MLD with standard therapy on the prevention of lymphedema after breast cancer surgery were included in our meta-analysis, which showed that MLD adds no benefit to the prevention of lymphedema compared to other interventions. This was because previous published reports of the effectiveness of preventing lymphedema were conflicting. On the one hand, in the studies by Cho et al and Zhang et al, the use of MLD had been related to a lower risk of developing lymphedema for breast cancer patients.^[21]^ On the other hand, Devoogdt et al compared the effect of exercise therapy and instructional guidelines with or without MLD to prevent lymphedema in patients after breast cancer surgery, and the results showed that there was no significant difference between the 2 groups at 6 months.^[20]^

### 4.2 Potential mechanisms

MLD is about pace, tension, and muscle as well as connective tissue compression, by means of the therapist's touch, which helps to improve circulation. MLD stimulates lymphatic and venous flow, enhances metabolism muscle tissue elasticity, and promotes relaxation by increasing parasympathetic nervous system activity and decreasing sympathetic nervous system activity. Such benefits, which were not assessed in the current study, can contribute to reducing anxiety and improving sleep and treatment adherence. However, multiple risk factors, including age, lymphedema onset, volume excess, number of infections, and obesity, can contribute to the failure of reducing limb volume after the treatment of breast cancer-related lymphedema.^[21]^ Therefore, comparable groups are needed to minimize possible selection biases. Although patients’ feelings of swelling improved after lymphedema treatment, it was important to choose a better treatment according to its outcome.

We noted moderate interstudy heterogeneity in our meta-analysis, which might result from variable clinical factors and clinical parameters. First, the technique, duration and frequency of MLD were not the same among the included studies. Second, the characteristics of the participants differed across the studies. For example, participants in the study by Sitzia et al were older than those in other trials.^[10]^ Third, the treatment in the comparison group was different among the studies, such as compression therapy and exercise strategies. Fourth, the assessments used for detecting the reduction of arm volume also differed among the studies, which might affect the comparison of the clinical outcomes.

### 4.3 Strengths and limitations

This updated meta-analysis of 17 studies provided consistent evidence of the equal effect of MLD and other treatments, which further confirmed and extended the preliminary findings of the 2 previous published meta-analyses. The first one published by Huang et al reported the addition of MLD to a standard treatment procedure, producing a non-significant effect on reducing arm volume for lymphedema related to breast cancer.^[14]^ They also reported that MLD had a non-significant effect on reducing the incidence of lymphedema in patients after breast cancer surgery. The other study performed by Ezzo et al found that MLD with or without compression therapy showed no significant improvement from baseline and no significant between-group differences for percent reduction.^[1]^ Despite the previously published meta-analysis demonstrating the effect of MLD on the treatment or prevention of lymphedema related to breast cancer surgery, the statistical power was limited since the sample sizes of the 2 meta-analyses were small (ranging from 426 to 566). To our knowledge, our study is the most comprehensive study with the largest sample size and without language limitations to evaluate the effectiveness of MLD in the treatment and prevention of lymphedema. Moreover, comprehensive and systematic search strategies were used to ensure the inclusion of almost all of the relevant RCTs and enabled us to minimize bias for conducting this meta-analysis and generate 17 studies and data from 1911 participants.

The largest sample size of this study allowed a detailed subgroup analysis to be conducted, and this subgroup analysis, such as research region, publication year, sample size, type of surgery, statistical analysis method, mean age, and the intervention time of association between MLD treatment and the development of lymphedema, was examined. Moreover, the careful estimation of methodological quality and a rigorous analysis method contributed to more strengthened and precise evidence concerning the effectiveness of MLD in the treatment and prevention of lymphedema after breast cancer surgery.

Nevertheless, a few limitations of our meta-analysis should be considered. First, only half of the studies included in our analysis reported adequate randomization in the study-group allocation, which could affect the treatment effect. Second, in 11 of the studies, the assessment staffs were not blinded to the measurement of the outcomes, which would lead to a certain bias of the pooled estimate. Third, some of the authors could not be contacted for retrieving the necessary data, and grey literature was not included in this meta-analysis, which could also lead to inaccurate results. Although there were several limitations in this meta-analysis, the clinical implication lied in that for breast cancer patients undergoing surgery, clinicians should consider the most effective treatment to minimize the development of lymphedema and improve the quality of life after breast cancer surgery.

## 5 Conclusion

In conclusion, our findings of this systematic review and meta-analysis provided evidence that MLD might not add any effect to the treatment and prevention of lymphedema after breast cancer surgery. However, it remains unclear whether MLD should be part of the treatment plan for breast cancer patients. Therefore, whether clinicians consider MLD for females with breast cancer in post-acute and long-term care requires further investigation because of the lack of solid supportive findings. Therefore, further well-designed and large-scale RCTs providing the highest level of evidence should be implemented to further test the evidence, especially in patients below the age of 60 years old or with an intervention time of 1 month.

## Author contributions

**Acquisition of data:** Lu Deng, Li He, Mining Liang.

**Analysis and interpretation of data:** Yongchao Hou, Zubin Mei, Mining Liang.

**Critical revision of the manuscript for important intellectual content:** all authors.

**Drafting of the manuscript:** Lezhi Li, Yang Zhang, Mining Liang.

**Study concept and design:** Mining Liang, Qiongni Chen, Kanglin Peng

**Study supervision:** Jincai Guo, Zubin Mei, Mining Liang.

**Conceptualization:** Mining Liang, Qiongni Chen, Kanglin Peng, Yang Zhang, Jincai Guo, Zubing Mei, Lezhi Li.

**Data curation:** Mining Liang, Lu Deng, Li He, Zubing Mei.

**Formal analysis:** Mining liang.

**Methodology:** Yongchao Hou.

**Software:** Mining Liang, Yongchao Hou.

**Supervision:** Jincai Guo, Zubing Mei, Lezhi Li.

**Writing – original draft:** Mining Liang, Yang Zhang, Lezhi Li.

**Writing – review & editing:** Mining Liang, Qiongni Chen, Kanglin Peng, Lu Deng, Li He, Yongchao Hou, Jincai Guo, Zubing Mei, Lezhi Li.

## Supplementary Material

Supplemental Digital Content
