# Supplementary material for: Manual lymphatic drainage for lymphedema in patients after breast cancer surgery: A systematic review and meta-analysis of randomized controlled trials
Source: Medicine (Baltimore). 2020 Dec 4;99(49):e23192. doi: 10.1097/MD.0000000000023192 (PMC7717855; doi:10.1097/MD.0000000000023192)
Supplement: Supplemental Digital Content [file medi-99-e23192-s001.docx]

**Supplementary Appendix．**

**Appendix**

**Search Strategy**

Search included: Pubmed, Embase, the Cochrane Library Central Register of Controlled Trials: till 2^nd^ May, 2019

**Table A1. Search strategy for PubMed (Publication date to 2019/05/02) result 107**

| 1. "Breast Neoplasms"[Mesh] |
| --- |
| 1. (breast OR mammary) [Title] |
| 1. (cancer* OR tumor* OR tumour* OR carcinom* OR neoplas* OR malignan* OR adenocarcinoma) [Title] |
| 1. 2 AND 3 |
| 1. 1 OR 4 |
| 1. "Lymphedema"[Mesh] |
| 1. (lymphoedema OR lymphedema OR oedema OR edema OR swelling OR elephantias* OR ‘lymphatic edema’) [Title] |
| 1. 6 OR 7 |
| 1. "Physical Therapy Modalities"[Mesh] |
| 1. "Drainage"[Mesh] |
| 1. (‘manual lymph*’ drainage OR physiotherapy OR ‘sequential pneumatic compression’ OR ‘complex decongestive therapy’ OR ‘decongestive lymphatic therapy’ OR ‘Foldi method’ OR ‘Vodder method’) [Title] |
| 1. 9 OR 10 OR 11 |
| 1. randomised controlled trial.pt |
| 1. randomized controlled trial.pt. |
| 1. controlled clinical trial.pt. |
| 1. (randomized OR randomized OR placebo OR randomly OR trial) [Title] |
| 1. 13 OR 14 OR 15 OR 16 |
| 1. 5 AND 8 AND 12 AND 17 |

**Table A2. Search strategy for Embase (Publication date to 2019/05/02) result 138**

| 1. 'breast tumor'/exp |
| --- |
| 2. ('breast'/exp OR breast OR mammary):ab,ti |
| 3. (cancer* OR tumor* OR tumour* OR carcinom* OR neoplas* OR malignan* OR adenocarcinoma):ab,ti |
| 4. #2 AND #3 |
| 5. #1 OR #4 |
| 6. 'lymphedema'/exp |
| 7. (lymphoedema OR lymphedema OR oedema OR edema OR swelling OR elephantias* OR 'lymphatic edema'):ab,ti |
| 8. #6 OR #7 |
| 9. 'physiotherapy'/exp |
| 10. 'drainage'/exp |
| 11. ('manual lymph*' AND drainage OR physiotherapy OR 'sequential pneumatic compression' OR 'complex decongestive therapy' OR 'decongestive lymphatic therapy' OR 'foldi method' OR 'vodder method'):ab,ti |
| 12. #9 OR #10 OR #11 |
| 13. 'randomized controlled trial'/exp |
| 14. (randomized OR placebo OR randomly OR trial):ab,ti |
| 15. #13 OR #14 |
| 16. #5 AND #8 AND #12 AND #16 |

**Table A3. Search strategy for Cochrane Library (Publication date to 2019/05/02)**

**result 209**

| 1. MeSH descriptor: [Breast Neoplasms] explode all trees |
| --- |
| 2. Breast OR Mammary:ti,ab,kw |
| 3. cancer* OR tumor* OR Tumour* OR Carcinom* OR Neoplas* OR Malignan* OR Adenocarcinoma:ti,ab,kw |
| 4. #2 and #3 |
| 5. #1 or #4 |
| 6. MeSH descriptor: [Lymphedema] explode all trees |
| 7. lymphoedema OR lymphedema OR oedema OR edema OR swelling OR elephantias* OR 'lymphatic edema':ti,ab,kw |
| 8. #6 or #7 |
| 9. MeSH descriptor: [Manual Lymphatic Drainage] explode all trees |
| 10. MeSH descriptor: [Physical Therapy Modalities] explode all trees |
| 11. 'manual lymph* drainage' OR physiotherapy OR 'sequential pneumatic compression' OR 'complex decongestive therapy' OR 'decongestive lymphatic therapy' OR 'foldi method' OR 'vodder method':ti,ab,kw |
| 12. #9 or #10 or #11 |
| 13. MeSH descriptor: [Randomized Controlled Trial] explode all trees |
| 14. MeSH descriptor: [Controlled Clinical Trial] explode all trees |
| 15. randomized OR placebo OR randomly OR trial:ti,ab,kw |
| 16. #13 or #14 or #15 |
| 17. #5 and #8 and #12 and #16 |

**Table A4.** Selection procedure of included and excluded studies

|  | **Reference list no.** |
| --- | --- |
| Studies included in the meta-analysis (n = 17) | 1-17 |
| Studies excluded from meta-analysis, and reasons are listed below (n = 10) | 18-27 |
| **Reason for exclusion** |  |
| No relevent outcomes recorded (n =3) | 18-20 |
| Letters, comments, reviews or meta-analyses (n =2) | 21,22 |
| Ineligible comparison (n =5) | 23-27 |

**REFERENCES LIST**

1. Andersen, L., I. Hojris, M. Erlandsen and J. Andersen (2000). "Treatment of breast-cancer-related lymphedema with or without manual lymphatic drainage--a randomized study." Acta Oncol 39(3): 399-405.
2. Belmonte, R., M. Tejero, M. Ferrer, J. M. Muniesa, E. Duarte, O. Cunillera and F. Escalada (2012). "Efficacy of low-frequency low-intensity electrotherapy in the treatment of breast cancer-related lymphoedema: a cross-over randomized trial." Clin Rehabil 26(7): 607-618.
3. Bergmann, A., M. G. da Costa Leite Ferreira, S. S. de Aguiar, R. de Almeida Dias, K. de Souza Abrahao, E. M. Paltrinieri, R. G. Martinez Allende and M. F. Andrade (2014). "Physiotherapy in upper limb lymphedema after breast cancer treatment: a randomized study." Lymphology 47(2): 82-91.
4. Cho, Y., J. Do, S. Jung, O. Kwon and J. Y. Jeon (2016). "Effects of a physical therapy program combined with manual lymphatic drainage on shoulder function, quality of life, lymphedema incidence, and pain in breast cancer patients with axillary web syndrome following axillary dissection." Support Care Cancer 24(5): 2047-2057.
5. Devoogdt, N., M. R. Christiaens, I. Geraerts, S. Truijen, A. Smeets, K. Leunen, P. Neven and M. Van Kampen (2011). "Effect of manual lymph drainage in addition to guidelines and exercise therapy on arm lymphoedema related to breast cancer: randomised controlled trial." Bmj 343: d5326.
6. Devoogdt, N., I. Geraerts, M. Van Kampen, T. De Vrieze, L. Vos, P. Neven, I. Vergote, M. R. Christiaens, S. Thomis and A. De Groef (2018). "Manual lymph drainage may not have a preventive effect on the development of breast cancer-related lymphoedema in the long term: a randomised trial." Br J Cancer 64(4): 245-254.
7. Didem, K., Y. S. Ufuk, S. Serdar and A. Zumre (2005). "The comparison of two different physiotherapy methods in treatment of lymphedema after breast surgery." Breast Cancer Res Treat 93(1): 49-54.
8. Johansson, K., M. Albertsson, C. Ingvar and C. Ekdahl (1999). "Effects of compression bandaging with or without manual lymph drainage treatment in patients with postoperative arm lymphedema." Lymphology 32(3): 103-110.
9. Johansson, K., E. Lie, C. Ekdahl and J. Lindfeldt (1998). "A randomized study comparing manual lymph drainage with sequential pneumatic compression for treatment of postoperative arm lymphedema." Lymphology 31(2): 56-64.
10. McNeely, M. L., D. J. Magee, A. W. Lees, K. M. Bagnall, M. Haykowsky and J. Hanson (2004). "The addition of manual lymph drainage to compression therapy for breast cancer related lymphedema: a randomized controlled trial." Breast Cancer Res Treat 86(2): 95-106.
11. Ridner, S. H., E. Poage-Hooper, C. Kanar, J. K. Doersam, S. M. Bond and M. S. Dietrich (2013). "A pilot randomized trial evaluating low-level laser therapy as an alternative treatment to manual lymphatic drainage for breast cancer-related lymphedema." Oncol Nurs Forum 40(4): 383-393.
12. Sitzia, J., L. Sobrido and W. Harlow (2002). "Manual lymphatic drainage compared with simple lymphatic drainage in the treatment of post-mastectomy lymphoedema: a pilot randomised trial." Physiotherapy 88(2): 99-107.
13. Szolnoky, G., B. Lakatos, T. Keskeny, E. Varga, M. Varga, A. Dobozy and L. Kemeny (2009). "Intermittent pneumatic compression acts synergistically with manual lymphatic drainage in complex decongestive physiotherapy for breast cancer treatment-related lymphedema." Lymphology 42(4): 188-194.
14. Tambour, M., M. Holt, A. Speyer, R. Christensen and B. Gram (2018). "Manual lymphatic drainage adds no further volume reduction to Complete Decongestive Therapy on breast cancer-related lymphoedema: a multicentre, randomised, single-blind trial."
15. Williams, A. F., A. Vadgama, P. J. Franks and P. S. Mortimer (2002). "A randomized controlled crossover study of manual lymphatic drainage therapy in women with breast cancer-related lymphoedema." Eur J Cancer Care (Engl) 11(4): 254-261.
16. Zhang, L., A. Fan, J. Yan, Y. He, H. Zhang, H. Zhang, Q. Zhong, F. Liu, Q. Luo, L. Zhang, H. Tang and M. Xin (2016). "Combining Manual Lymph Drainage with Physical Exercise after Modified Radical Mastectomy Effectively Prevents Upper Limb Lymphedema." Lymphat Res Biol 14(2): 104-108.
17. Zimmermann, A., M. Wozniewski, A. Szklarska, A. Lipowicz and A. Szuba (2012). "Efficacy of manual lymphatic drainage in preventing secondary lymphedema after breast cancer surgery." Lymphology 45(3): 103-112.
18. de Oliveira, M. M., L. F. de Rezende, M. T. do Amaral, M. P. Pinto e Silva, S. S. Morais and M. S. Gurgel (2014). "Manual lymphatic drainage versus exercise in the early postoperative period for breast cancer." Physiother Theory Pract 30(6): 384-389.
19. Freire de Oliveira, M. M., M. S. Costa Gurgel, M. T. Pace do Amaral, B. J. Amorim, C. D. Ramos, J. G. Almeida Filho, L. F. de Rezende and L. O. Zanatta Sarian (2017). "Manual Lymphatic Drainage and Active Exercise Effects on Lymphatic Function Do Not Translate Into Morbidities in Women Who Underwent Breast Cancer Surgery." Arch Phys Med Rehabil 98(2): 256-263.
20. Martin, M. L., M. A. Hernandez, C. Avendano, F. Rodriguez and H. Martinez (2011). "Manual lymphatic drainage therapy in patients with breast cancer related lymphoedema." BMC Cancer 11: 94.
21. Ezzo, J., E. Manheimer, M. L. McNeely, D. M. Howell, R. Weiss, K. I. Johansson, T. Bao, L. Bily, C. M. Tuppo, A. F. Williams and D. Karadibak (2015). "Manual lymphatic drainage for lymphedema following breast cancer treatment." Cochrane Database Syst Rev(5): Cd003475.
22. Huang, T. W., S. H. Tseng, C. C. Lin, C. H. Bai, C. S. Chen, C. S. Hung, C. H. Wu and K. W. Tam (2013). "Effects of manual lymphatic drainage on breast cancer-related lymphedema: a systematic review and meta-analysis of randomized controlled trials." World J Surg Oncol 11: 15.
23. Castro-Sanchez, A. M., C. Moreno-Lorenzo, G. A. Mataran-Penarrocha, M. E. Aguilar-Ferrandiz, I. Almagro-Cespedes and J. Anaya-Ojeda (2011). "[Preventing lymphoedema after breast cancer surgery by elastic restraint orthotic and manual lymphatic drainage: a randomized clinical trial]." Med Clin (Barc) 137(5): 204-207.
24. Dayes, I. S., T. J. Whelan, J. A. Julian, S. Parpia, K. I. Pritchard, D. P. D'Souza, L. Kligman, D. Reise, L. LeBlanc, M. L. McNeely, L. Manchul, J. Wiernikowski and M. N. Levine (2013). "Randomized trial of decongestive lymphatic therapy for the treatment of lymphedema in women with breast cancer." J Clin Oncol 31(30): 3758-3763.
25. Torres Lacomba, M., M. J. Yuste Sanchez, A. Zapico Goni, D. Prieto Merino, O. Mayoral del Moral, E. Cerezo Tellez and E. Minayo Mogollon (2010). "Effectiveness of early physiotherapy to prevent lymphoedema after surgery for breast cancer: randomised, single blinded, clinical trial." Bmj 340: b5396.
26. Uzkeser, H., S. Karatay, B. Erdemci, M. Koc and K. Senel (2015). "Efficacy of manual lymphatic drainage and intermittent pneumatic compression pump use in the treatment of lymphedema after mastectomy: a randomized controlled trial." Breast Cancer 22(3): 300-307.
27. Oliveira, M. M. F., M. S. C. Gurgel, B. J. Amorim, C. D. Ramos, S. Derchain, N. Furlan-Santos, C. C. Dos Santos and L. O. Sarian (2018). "Long term effects of manual lymphatic drainage and active exercises on physical morbidities, lymphoscintigraphy parameters and lymphedema formation in patients operated due to breast cancer: A clinical trial." PLoS One 13(1): e0189176.

Table A5: Subgroup analyses of the effect of MLD treatment of lymphedema in 8 RCTs

| Variables | SMD | 95% CI | Degree of heterogeneity (I2 statistics; %) | *P* | No. of included Studies | *P^a^* |
| --- | --- | --- | --- | --- | --- | --- |
| Total | -0.09 | -0.85 to 0.67 | 91.3 | <0.001 | 8 |  |
| Region |  |  |  |  |  | <0.001 |
| European | -0.65 | -1.54 to 0.24 | 89.9 | <0.001 | 5 |  |
| America | -0.02 | -0.41 to 0.37 | 0 | 0.069 | 2 |  |
| Publication year |  |  |  |  |  | <0.001 |
| >2010 | -0.01 | -0.35 to 0.34 | 0 | 0.693 | 2 |  |
| 2000-2010 | -0.16 | -1.88 to 1.56 | 95.7 | <0.001 | 4 |  |
| <2000 | 0.07 | -0.44 to 0.58 | 0 | 0.383 | 2 |  |
| Sample size |  |  |  |  |  | <0.001 |
| Between 20 to 30 | -0.56 | -1.59 to 0.47 | 88.6 | <0.001 | 3 |  |
| Less than 20 | 0.32 | -1.38 to 2.02 | 95.0 | <0.001 | 4 |  |
| Surgery type |  |  |  |  |  | <0.001 |
| Mastectomy | -0.54 | -1.69 to 0.60 | 92.4 | <0.001 | 3 |  |
| Mastectomy + axillary clearance | 3.00 | 1.90 to 4.11 | 84.2 | - | 1 |  |
| Data analysis |  |  |  |  |  | <0.001 |
| ITT | -0.56 | -1.53 to 0.42 | 89.2 | <0.001 | 3 |  |
| PP | 0.25 | -1.00 to 1.50 | 93.5 | <0.001 | 5 |  |
| Mean age |  |  |  |  |  | <0.001 |
| More than 60 | 0.87 | -0.44 to 2.18 | 92.4 | <0.001 | 3 |  |
| Less than 60 | -1.77 | -2.23 to -1.31 | 0 | 0.952 | 2 |  |
| Time |  |  |  |  |  | <0.001 |
| 3 months | -1.77 | -2.23 to -1.30 | 0 | 0.952 | 2 |  |
| 4 weeks | 0.11 | -0.22 to 0.44 | 0 | 0.816 | 3 |  |
| <4 weeks | 0.85 | -0.67 to 2.37 | 92.5 | <0.001 | 3 |  |


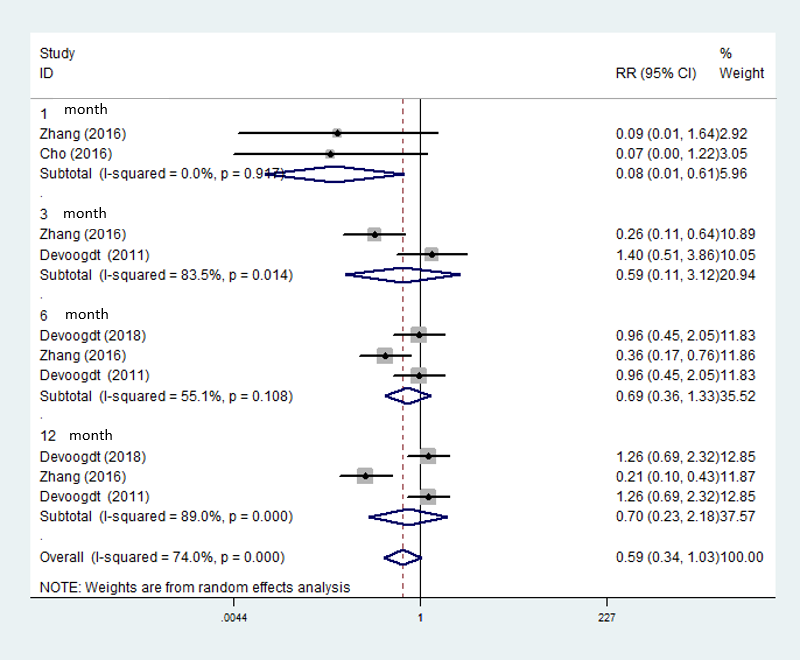


Figure A2 Stratiﬁed Analyses for the Association Between manual lymph drainage (MLD) Treatment and Risk of post-mastectomy lymphedema in 4 randomized controlled trial (RCTs)
